# Late Gadolinium Enhancement Entropy as a Novel Imaging Biomarker of Myocardial Tissue Heterogeneity—A Comprehensive Review

**DOI:** 10.3390/diagnostics16172735

**Published:** 2026-08-26

**Authors:** Apostolos Vrettos, Michael A. Winkler, Alexios Antonopoulos, Maria Prasinou, Uzma Gul, Polyvios Demetriades, Sanjeev Bhattacharyya

**Affiliations:** 1 Unit of Inherited Cardiac Conditions and Sports Cardiology, First Department of Cardiology, Athens Medical School, National and Kapodistrian University of Athens, 115 27 Athens, Greece; 2 Hygeia Hospital, 151 21 Marousi, Greece; 3 Department of Radiology and Imaging Sciences, Emory University School of Medicine, Atlanta, GA 30322, USA; michael.winkler@emory.edu; 4 Department of Physiology, Medical School, National and Kapodistrian University of Athens, 115 27 Athens, Greece; 5 University Hospitals Coventry and Warwickshire NHS Trust, Clifford Bridge Road, Coventry CX2 2DX, UK; uzma.gul2@nhs.net; 6 Barts Heart Centre, St Bartholomew’s Hospital, Barts Health NHS Trust, London EX1A 7BE, UKsanjeev.bhattacharyya@nhs.net (S.B.)

**Keywords:** cardiovascular magnetic resonance, LGE entropy, myocardial heterogeneity, scar heterogeneity, ventricular tachyarrhythmia, sudden cardiac death, texture analysis, radiomics

## Abstract

Late gadolinium enhancement (LGE) cardiac magnetic resonance is the reference standard for non-invasive myocardial tissue characterization. Conventional LGE analysis focuses on the presence and extent of fibrosis, yet these measures incompletely describe the spatial complexity of myocardial scar that underpins arrhythmogenesis. Entropy, derived from radiomic analysis of LGE signal-intensity distributions, has emerged as a surrogate marker of myocardial tissue heterogeneity. Mechanistically, heterogeneous fibrosis promotes electrical conduction alterations, and entropy serves as a global descriptor of this complex substrate. A growing body of evidence suggests that higher LGE entropy is associated with increased arrhythmogenicity and major adverse cardiac events. Several studies have shown that this association remains significant after adjustment for conventional clinical and imaging predictors. A smaller number of studies have gone further, demonstrating that incorporation of LGE entropy improves the discriminatory or reclassification performance of established risk-prediction models. This narrative review critically synthesizes the current evidence on LGE-derived entropy, compares methodological approaches and clinical applications, and discusses its principal limitations. After standardization and prospective validation, entropy-based phenotyping may prove useful for individualized risk stratification beyond conventional LGE metrics and guide clinical decision-making.

## 1. Introduction

Recent cardiac histopathological and electrophysiological studies have demonstrated that heterogeneous or textured scar architecture is more likely to result in conduction slowing and re-entry than homogeneous regions of fibrosis. An increasing body of complementary cardiac magnetic resonance (CMR) evidence suggests that high spatial frequency abnormality in late gadolinium enhancement (LGE) signal-intensity, a radiomic marker, may be valuable for the purpose of risk stratification beyond macroscopic scar burden alone. Radiomic analysis is a common method applied to medical imaging that evaluates hard-to-perceive features such as first-order histogram characteristics, signal distribution, gradients, correlations between adjacent pixels (texture analysis), and degree of randomness in magnetic resonance imaging (MRI) signal-intensity [1,2,3]. These radiomic techniques are used to evaluate MRI findings across a wide range of conditions, including breast cancer, neurological disorders, and liver fibrosis [1,4,5]. “Entropy,” a foundational term of art in the practice of radiomics, is a statistical feature of LGE seen on CMR studies that reflects the randomness or variability of signal-intensities within abnormal myocardium. Entropy may therefore capture aspects of the internal organization of fibrotic tissue and provide a more threshold-independent framework for quantifying tissue heterogeneity.

Although an increasing number of studies have investigated LGE entropy in cardiovascular disease, the available evidence remains fragmented across different disease populations, methodological approaches, and clinical endpoints. It remains unclear whether entropy is disease-specific imaging marker or reflects a broader phenotype of myocardial substrate heterogeneity with prognostic relevance across different cardiovascular conditions. Distinctions between whole-myocardial and scar-specific entropy, as well as between first-order histogram entropy and more complex second-order texture-based approaches, need clarification and elucidation. This narrative review aims to summarize the available evidence, critically integrate findings across cardiovascular diseases, compare the principal methodological approaches to entropy assessment, and examine whether entropy provides clinically relevant information beyond conventional measures of LGE presence and extent in cardiovascular risk stratification.

## 2. Review Methodology

This narrative review was conducted to provide a critical overview of the current evidence regarding CMR-derived LGE entropy for risk stratification and outcome prediction in cardiovascular disease. A literature search was performed using PubMed/MEDLINE, focusing on studies published up to 31 December 2025. Search terms included combinations of “cardiac magnetic resonance”, “late gadolinium enhancement”, “LGE”, “entropy”, “texture analysis”, “ventricular arrhythmia”, “sudden cardiac death” and “risk stratification”. Additional relevant studies were identified through manual screening of the reference lists of retrieved articles. Studies were considered relevant if they investigated entropy derived from LGE-CMR imaging and evaluated its relationship with arrhythmic substrates, cardiovascular outcomes, or risk stratification. Studies were synthesized narratively according to the underlying cardiovascular disease and principal clinical or prognostic application. Given the heterogeneity in study populations, imaging acquisition and post-processing protocols, entropy definitions, and clinical endpoints, no quantitative meta-analysis was undertaken.

Because the included studies evaluated clinically heterogeneous outcomes, endpoints were grouped into distinct clinical domains: arrhythmic events, including sustained ventricular tachycardia (VT), ventricular fibrillation (VF), sudden cardiac death (SCD), or aborted SCD; device-treated ventricular arrhythmias, defined as appropriate implantable cardioverter-defibrillator (ICD) therapy with anti-tachycardia pacing or shock; heart-failure-related outcomes, including hospitalization, cardiac transplantation, or ventricular assist device implantation; mortality; and broader composite cardiovascular outcomes, including major adverse cardiovascular events (MACEs). Appropriate ICD therapy was treated as a surrogate for clinically significant ventricular arrhythmia rather than as equivalent to SCD. Because these endpoints differ substantially in clinical significance, they were interpreted separately in the narrative synthesis.

## 3. Assessment of LGE Entropy on Cardiac MRI

Visual estimation and the standard deviation approach are the two most widely used methods for quantifying LGE [6,7]. Visual estimation is inherently subjective and observer-dependent, so its accuracy cannot always be ensured. In contrast, the standard deviation method uses a semi-quantitative scoring system. LGE is defined by signal-intensity thresholds set at a certain number of standard deviations above the mean intensity of normal myocardium, typically in the range of 2–6 standard deviations, and expressed as a percentage of total left ventricular mass [8]. However, both techniques primarily capture the extent of LGE rather than its internal complexity. Moreover, conventional LGE metrics rely on standard approaches utilizing signal cut-offs, which may vary between scanners and protocols.

**Figure 1:**
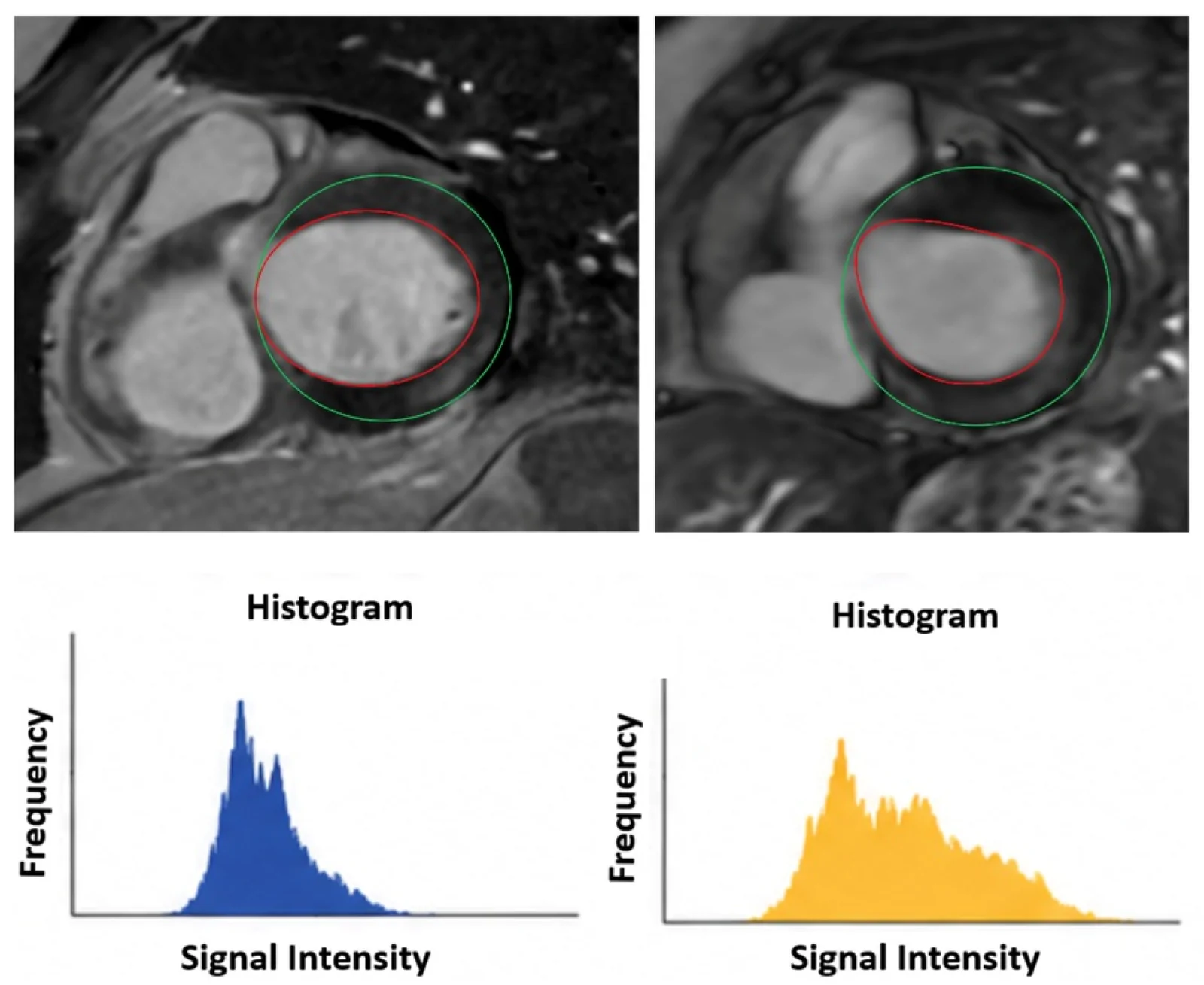
Histograms of left ventricular myocardial signal-intensity from two patients with subepicardial LGE. Patient 2 experienced a greater burden of ventricular arrhythmias. Patient 1 demonstrates a narrow signal-intensity distribution with a high concentration of pixels, consistent with relatively homogeneous myocardial tissue. In contrast, Patient 2 exhibits a broader, more dispersed distribution of signal-intensities, consistent with increased image heterogeneity and potentially greater myocardial tissue heterogeneity.

To provide a quantitative assessment of signal-intensity heterogeneity while reducing dependence on binary signal-intensity thresholds, entropy analysis has been applied to LGE-CMR. Entropy analysis reduces dependence on binary signal-intensity thresholds by using the full myocardial signal distribution Most studies calculate entropy using LGE short-axis stacks after segmentation of the left ventricular myocardium. Pixel intensity distributions are extracted from the myocardium or from defined scar regions and entropy values are computed from their probability distributions (Figure 1).

Entropy can be derived using different radiomic approaches. First-order entropy is calculated from the histogram of voxel intensities within the region of interest and quantifies the randomness of signal-intensity values without considering their spatial arrangement. First-order entropy is most commonly calculated using the following formula:

$$H=-\sum_{i=1}^{N}p_{i}\log_{b}\left(p_{i}\right),$$

where $p_{i}$ represents the probability of observing signal-intensity value or grey-level bin i within the region of interest, N is the number of possible intensity levels or bins, and b is the logarithm base. The probabilities are obtained by normalizing the signal-intensity histogram so that $\sum_{i}p_{i}=1$. A homogeneous region dominated by a small number of signal-intensity values has relatively low entropy, whereas a broader and less predictable intensity distribution results in higher entropy. First-order entropy depends only on the frequency distribution of signal-intensities and does not encode voxel location, adjacency, connectivity, or spatial arrangement. It may therefore serve as a surrogate marker of underlying myocardial tissue heterogeneity without directly measuring spatial tissue organization.

In contrast, second-order entropy is calculated from texture matrices that explicitly incorporate spatial relationships between voxels. The most commonly used approach is the grey-level co-occurrence matrix (GLCM), in which $P\left(i,j\right)$ represents the normalized probability that grey levels i and j occur in a specified spatial relationship. GLCM entropy can be expressed as

$$H_{\mathrm{GLCM}}=-\sum_{i}\sum_{j}P\left(i,j\right)\log_{b}\left[P\left(i,j\right)\right].$$

Because the GLCM incorporates neighbouring voxel relationships, second-order entropy provides information about local textural organization and spatial heterogeneity. Although both orders of entropy quantify tissue heterogeneity, they are not interchangeable and may provide complementary biological information. Importantly, the majority of studies reviewed here employed first-order histogram entropy, whereas second-order entropy has been evaluated mainly in exploratory radiomics studies using machine learning.

Both whole-myocardium and scar can be measured for entropy. The first case evaluates signal heterogeneity across the entire left ventricular myocardium in contrast-enhanced images, whereas the latter quantifies heterogeneity specifically within LGE-defined scar regions [9]. An entirely homogeneous cardiac signal would theoretically result in a low-entropy CMR image, whereas higher entropy values reflect broader or more complex distributions of signal-intensity. Accordingly, entropy values should be interpreted in the context of both the computational method and the anatomical region from which they were derived.

## 4. Image Entropy as a Novel Marker of Arrhythmic Risk

**Figure 2:**
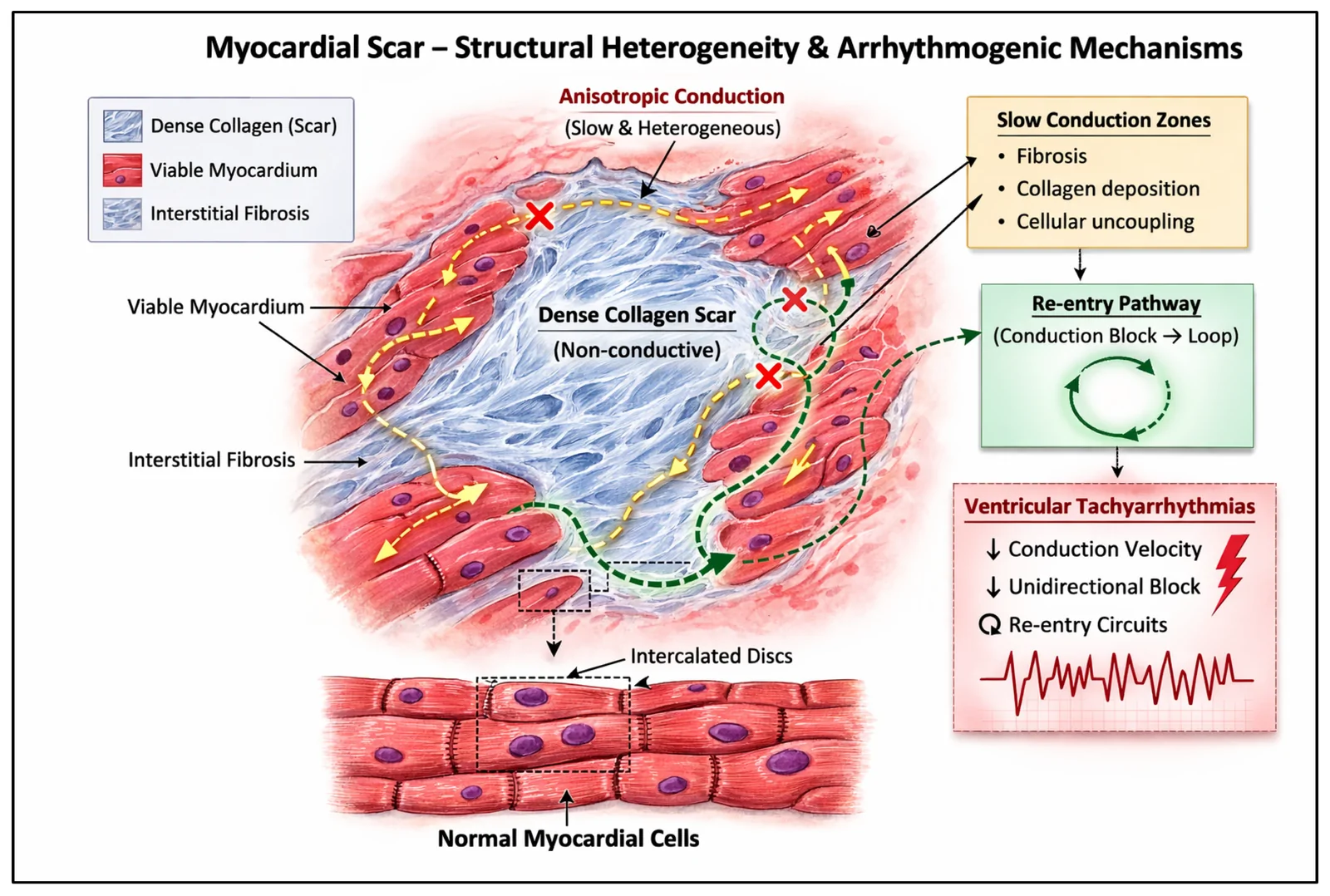
Structural heterogeneity of myocardial scar and arrhythmogenic mechanisms.

The presence of fibrosis causing electric instability in the left ventricle (LV) myocardium through re-entrant arrhythmias is a well-recognized mechanism for SCD [10]. Histologically, myocardial scar comprises dense collagen, often termed as “core scar,” surrounded by interspersed viable myocardium and fibrosis, collectively referred to as the “grey zone.” This structural heterogeneity creates anisotropic conduction, slow conduction zones and re-entry pathways that predispose to ventricular as well as atrial tachyarrhythmias (Figure 2). The traditional way of defining “core scar” and “grey zone” areas, typically depending on signal-intensity thresholds, may be suboptimal as a metric due to variation in definition, with studies evaluating scar size as a predictor of cardiac events yielding inconsistent findings. For instance, some CMR investigations have identified both the grey zone and core scar as independent predictors of cardiovascular events [11,12], whereas others have reported predictive value for only the core scar [13], or only the grey zone [14,15], with respect to arrhythmic outcomes. These discrepancies may be due in part to unmeasured myocardial heterogeneity. First-order LV entropy does not directly encode spatial arrangement, but it is derived from the full signal-intensity distribution independently of predefined thresholds and may be sensitive to gradual variations in myocardial signal-intensity. Consequently, it can serve as a surrogate marker of underlying tissue heterogeneity.

### 4.1. Supporting Mechanistic and Computational Evidence from Atrial Fibrosis Studies

The proarrhythmic impact of heterogeneous scar has been established in both clinical investigations and computational models. Simulations of fibrotic myocardium have shown that heterogeneous scar patterns promote conduction block and re-entry [10]. In these models, complex spatial patterns of fibrosis, analogous to higher entropy, were associated with greater electrical instability. Specifically, complex scar geometries with irregular interfaces and fragmented components facilitated re-entrant pathways. Although entropy was not the sole predictor, it contributed to distinguishing high-risk patterns. These findings reinforce the concept that structural complexity, rather than scar quantity alone, drives arrhythmic risk. Zahid et al. [16] reconstructed personalized 3D atrial models with individualized fibrosis patterns from LGE-CMR. Dynamic pacing was able to induce atrial fibrillation (AF) in atrial models that included sufficient amounts of fibrosis. Further simulations showed that AF was perpetuated by re-entrant drivers localized in boundary zones between fibrotic and non-fibrotic tissue, characterized by high fibrosis entropy and fibrosis density. Subsequently, Ali et al. [17] showed that successful ablation lesions turn a “bad” fibrosis distribution (one characterized with high entropy and density) into a “good” fibrosis distribution (characterized with low entropy). Suzuki et al. [18] evaluated atrial LGE entropy in areas with and without electrically fractionated potentials—which may be serving as substrates of AF. Areas containing electrically fractionated potentials demonstrated significantly higher LGE entropy values than non-fractionated regions. Although these studies do not directly validate first-order LGE histogram entropy, they provide supporting biological and computational evidence that greater heterogeneity and spatial organization of fibrosis contribute to atrial arrhythmogenic substrates.

### 4.2. Clinical Evidence for Ventricular Arrhythmia Risk

The prognostic significance of LGE entropy across different cardiac conditions will be discussed in more detail in the relevant sections of this review. From the perspective of arrhythmic risk stratification, however, a substantial body of evidence demonstrates an association between higher LGE signal-intensity heterogeneity and an increased propensity for ventricular arrhythmias, irrespective of the underlying disease substrate. For example, in addition to the percentage of grey and core scar zone on LGE-CMR, texture analysis metrics and grey-zone entropy quantification have been linked to VT/VF attacks and cardiac mortality in heart failure (HF) patients [19]. In another study, in patients with and without ischemic heart disease presenting with ventricular arrhythmias, LV LGE entropy remained independently associated with MACEs, defined by all-cause death, incident VAs requiring therapy, or HF hospitalization, on top of LVEF and LGE extent, and helped with risk stratification even in cases without visible myocardial scar [20]. Supporting a possible mechanistic link between tissue heterogeneity and electromechanical instability, Zhao et al. reported that LGE-derived entropy correlated with three-dimensional LV mechanical dispersion in HCM [21].

### 4.3. Device-Treated Ventricular Arrhythmias and Potential ICD Applications

Entropy analysis has also been investigated in relation to device-treated ventricular arrhythmias. Gould et al. reported that higher scar mean entropy was associated with appropriate ICD therapy [22]. A subsequent study by Gould et al. [23] examined anti-tachycardia pacing (ATP) response and found that patients in whom ATP was unsuccessful, before the delivery of an effective ICD shock, had higher mean entropy than those whose ventricular arrhythmias were successfully terminated by ATP, suggesting that a higher degree of scar signal-intensity heterogeneity may be associated with more refractory ventricular arrhythmias less amenable to ATP. These findings raise the possibility that entropy could eventually support more individualized ICD management, including improved counselling regarding the likelihood of shock therapy, refinement of ATP programming, and more informed device selection, such as the choice between transvenous and subcutaneous ICD systems. Although these applications have not yet been prospectively validated, the observed association between higher entropy and ATP failure provides a clinically relevant signal that merits further investigation in dedicated prospective studies. At present, however, no validated entropy-based threshold exists to guide ICD implantation, device-type selection, or programming, and whether such strategy would improve clinical outcomes. Importantly, appropriate ICD therapy should be regarded as a surrogate arrhythmic outcome rather than a direct equivalent of SCD because its occurrence is influenced by device detection thresholds, programmed therapy zones, and treatment algorithms. Accordingly, associations with appropriate ICD therapy should be interpreted as reflecting susceptibility to device-treated ventricular arrhythmias rather than direct prediction of SCD.

### 4.4. Beyond Arrhythmic Risk

Beyond arrhythmic and device-treated outcomes, whole-LV LGE entropy has also been associated with broader cardiovascular endpoints. Zhang et al. reported that higher LV entropy was independently associated with MACEs in patients with hypertension and high cardiovascular risk [24], despite only modest correlation with LGE burden, suggesting that it may provide information about myocardial signal-intensity heterogeneity not fully represented by conventional scar quantification. Moreover, many studies have shown that the risk of MACEs increases progressively with higher entropy values, especially when measured for the whole LV [25,26,27]. These findings extend the potential relevance of entropy beyond arrhythmic risk, although composite cardiovascular endpoints should be interpreted separately from ventricular arrhythmias and SCD.

## 5. Ischemic Heart Disease and Myocardial Infarction

Among patients with prior myocardial infarction, LGE entropy has been proposed as a threshold-independent measure of myocardial heterogeneity and may confer prognostic information (Table S1).

The first clinical evidence supporting the prognostic value of LGE entropy was provided by Androulakis et al. [28], who introduced entropy as a threshold-independent measure of signal-intensity heterogeneity in post-MI patients undergoing primary prevention ICD implantation. Unlike conventional scar quantification, which relies on predefined signal-intensity thresholds, entropy characterizes the distribution of signalintensities within the myocardium. This study demonstrated that higher scar entropy was independently associated with device-treated ventricular arrhythmias, defined by appropriate ICD therapy with ATP or shock for monomorphic VT or VF, after adjustment for clinical variables, scar grey zone, and scar transmurality, while entropy of the entire LV independently predicted all-cause mortality. These findings suggested that scar entropy may indirectly reflect features of the local arrhythmogenic substrate, whereas global LV entropy may capture more diffuse adverse myocardial remodelling [28].

Subsequent studies evaluated different and partly composite outcomes. Wang et al. demonstrated that entropy derived from the peri-infarct-border zone and the combined infarct-border zone independently predicted MACEs comprising cardiac death, heart failure, recurrent non-fatal MI, stroke, ventricular arrhythmia, and severe-angina rehospitalization and improved risk stratification beyond LVEF [29]. Zhao et al. evaluated a composite that included SCD and sustained ventricular arrhythmia together with ICD-treated events, whereas the later Zhao study additionally incorporated heart-failure events [30,31]. These findings support the concept that border zone signal-intensity heterogeneity may reflect the presence of arrhythmogenic substrate following myocardial infarction.

More recent investigations have expanded entropy analysis beyond infarct-specific assessment toward more global characterization of myocardial heterogeneity. In a multicentre study of patients with coronary artery disease, Wang et al. demonstrated that whole-LV entropy independently predicted MACEs after adjustment for conventional clinical variables, LGE burden, native T1 and extracellular volume, suggesting that entropy captures information complementary to established CMR tissue-characterization techniques [32]. Consistent with this concept, Li et al. showed that whole-LV entropy and scar entropy provide complementary prognostic information, with LV entropy independently associated with MACEs, whereas scar entropy remained more strongly associated with ventricular arrhythmias [33].

More advanced radiomic analyses have evaluated first-order entropy alongside spatially informed morphological and texture features. Zaidi et al. incorporated peri-infarct Shannon entropy into a machine-learning framework together with measures including scar components, interface area, gradient, and local binary patterns, demonstrating improved prediction of a more specifically arrhythmic endpoint comprising SCD, aborted SCD, or hemodynamically unstable VT, compared with guideline-based clinical models [34]. This provides more direct evidence linking infarct-related imaging characteristics with severe arrhythmic outcomes. These findings suggest that entropy is unlikely to represent a standalone biomarker but rather one component of a broader quantitative assessment of infarct architecture.

Collectively, the available evidence from studies evaluating first-order entropy in post-MI patients demonstrates an association between greater LGE heterogeneity and adverse clinical outcomes. Scar- and peri-infarct entropy have been associated with both device-treated ventricular arrhythmias and harder arrhythmic outcomes, whereas whole-LV entropy has predominantly been linked to mortality or broader composite cardiovascular events. Although the evidence is remarkably consistent in demonstrating prognostic value, most studies remain retrospective and observational, highlighting the need for prospective multicentre validation and methodological standardization before entropy can be incorporated into routine post-MI risk stratification.

## 6. Hypertrophic Cardiomyopathy

The histopathological changes observed in hypertrophic cardiomyopathy (HCM) include replacement fibrosis resulting from microvascular ischemia, myofibrillar disarray caused by sarcomeric gene mutations, and increased deposition of extracellular collagen. In patients with HCM, VT is believed to originate in regions containing a mixture of dense fibrotic scar and surviving disorganized myocytes, creating a substrate for re-entrant arrhythmias [35]. Late gadolinium enhancement on CMR is widely used to identify replacement fibrosis in hypertrophic cardiomyopathy (HCM) and has been consistently associated with ventricular arrhythmias, SCD, and adverse cardiovascular outcomes [36].

Initial evidence focused on entropy measured within LGE-defined scar (Table S2). In a retrospective single-centre study of 68 patients with HCM and visible LGE, Ye et al. found that higher scar entropy was independently associated with a heterogeneous ventricular-arrhythmia phenotype comprising previous aborted cardiac arrest, sustained VT and NSVT after adjustment for LVEF, LGE extent, maximal wall thickness, and left atrial diameter [9]. In contrast, whole-LV entropy was not independently associated with arrhythmias in this cohort, suggesting that signal-intensity heterogeneity within LGE-defined scar may be more closely related to the arrhythmogenic substrate than global myocardial signal-intensity distribution. Nevertheless, this finding should be interpreted cautiously because the study included only patients with visible scar, classified ventricular arrhythmias largely using 24 h ambulatory monitoring and prior clinical records, and involved a relatively small number of patients from a single centre.

Subsequent studies evaluated entropy across the entire LV myocardium and examined broader clinical outcomes. In a prospective cohort of 109 patients, Zhao et al. [26] reported that whole-LV LGE entropy was independently associated with a composite of SCD, heart-failure hospitalization, and appropriate ICD therapy. In receiver-operating-characteristic analysis, LGE entropy demonstrated the highest numerical discrimination for the composite endpoint, with an AUC of 0.893, compared with 0.826 for LGE mass%, 0.716 for the 2020 AHA/ACC guideline-based risk classification, and 0.610 for LVEF. Pairwise DeLong testing confirmed significantly greater discrimination for LGE entropy than for the AHA/ACC classification and LVEF, although its performance was not significantly different from that of LGE mass% [26]. Although these findings do not necessarily establish superiority over conventional LGE burden, they do suggest that entropy may provide risk information beyond ventricular systolic function and conventional guideline-based classification.

Evidence about the incremental role of entropy to LGE burden comes from Gu et al. [27]. In their retrospective cohort of 337 patients with HCM, the investigators calculated first-order entropy from the entire LV myocardium on LGE images. During follow-up, 33 patients experienced the primary endpoint of heart-failure readmission, while 43 experienced the broader secondary composite endpoint, which included heart-failure readmission and death. Patients with events had substantially higher whole-LV entropy than those without events. In multivariable Cox regression, both entropy and conventional LGE extent remained independently associated with the primary and secondary endpoints (HR for entropy of 1.291 and 1.273, respectively). Adding entropy to models containing age, left atrial diameter, and LGE improved discrimination and reclassification (final C-indices of 0.872 and 0.838 for the primary and secondary endpoints, respectively). These findings suggest that whole-LV entropy provides information complementary to conventional LGE burden and may reflect broader myocardial signal-intensity heterogeneity associated with heart-failure progression. It is worth noting the limitations of the mentioned studies. In Zhao et al. [26], the analysis was based on only 21 composite events among 109 patients, including three SCD, nine ICD therapies for ventricular arrhythmias, and nine cases of new heart failure. In Gu et al. [27], the observed outcomes were predominantly heart-failure-related, which may not be interpreted as direct evidence of improved sudden-death or ventricular-arrhythmia prediction. Furthermore, the study was retrospective, had relatively few events, lacked external validation and included limited T1-mapping data, preventing a comprehensive comparison between entropy and extracellular volume. Validation in larger independent cohorts is required.

Evidence from the multicentre NHLBI HCM Registry provides additional mechanistic support but should be distinguished from longitudinal outcome studies. Among 1736 patients, Antiochos et al. [25] found that whole-LV entropy was associated with sarcomere mutation status, biomarkers of myocardial injury and hemodynamic stress, wall thickness, LGE, extracellular volume, myocardial strain, and the ESC sudden-death risk score. Entropy was also the strongest independent predictor of VT on Holter monitoring, including among patients without visually detectable LGE. This study evaluated associations between entropy, baseline risk markers, and prevalent Holter-detected ventricular arrhythmias rather than prospectively adjudicated incident events; therefore, the independent prospective predictive value of entropy remains unestablished.

Collectively, higher first-order entropy derived from LGE-CMR is associated with adverse HCM phenotypes and clinical outcomes. Scar entropy may be more closely associated with localized arrhythmic risk, whereas whole-LV entropy may reflect broader myocardial abnormalities associated with both Holter-detected ventricular arrhythmias and heart-failure-related outcomes. It should, however, be noted that neither of those first-order measures directly encodes the spatial arrangement, connectivity, or geometry of myocardial fibrosis. Their associations with ventricular arrhythmias and adverse outcomes should therefore be interpreted as evidence that signal-intensity heterogeneity may indirectly reflect an adverse myocardial substrate rather than as direct measurement of scar spatial complexity. The evidence remains observational, with heterogeneous endpoints, limited event numbers, and no externally validated thresholds. Prospective multicentre studies using standardized methods and adjudicated clinical outcomes are needed to establish incremental value beyond current HCM risk models.

## 7. Non-Ischemic Cardiomyopathy

### 7.1. Dilated Cardiomyopathy

Non-ischemic dilated cardiomyopathy (DCM) is a diverse myocardial disorder clinically presenting with heart failure and an increased risk of ventricular arrhythmias and SCD. It is marked by a broad spectrum of structural and pathological alterations, including enlargement of the ventricular chambers, thinning of the ventricular walls, and the development of myocardial fibrosis. At the histological level, myocardial interstitial fibrosis in patients with non-ischemic cardiomyopathy exhibits considerable variability. Several distinct fibrotic patterns, including diffuse microscopic scarring, perivascular fibrosis, perimysial fibrosis, and endomysial fibrosis, may be observed either across different individuals or within the same individual as the disease progresses [37,38]. These diverse microscopic forms of fibrosis are likely associated with different degrees and types of myocardial remodelling, which may translate into varying susceptibilities to adverse clinical outcomes [39,40]. Consequently, emerging imaging biomarkers capable of characterizing the heterogeneity of fibrosis could provide important prognostic information and enhance risk stratification in patients with dilated cardiomyopathy (DCM), which is especially important when considering candidacy for ICD therapy [41]. Evidence in DCM has evaluated both whole-LV and scar-specific entropy (Table S3). Muthalaly et al. reported that higher whole-LV entropy derived from LGE-CMR was independently associated with a composite arrhythmic endpoint, comprising appropriate ICD therapy, sustained ventricular arrhythmia, and SCD [42].

Hammersley et al. subsequently evaluated entropy specifically within LGE-defined fibrotic regions, including scar core, grey zone, and combined fibrosis, in a non-ischemic cardiomyopathy cohort comprising predominantly patients with DCM [43]. Higher fibrosis entropy was associated with a harder arrhythmic endpoint comprising SCD, aborted SCD, and sustained VT after adjustment for LVEF and NYHA class and remained associated with events after accounting for fibrosis mass. Integration of fibrosis presence with entropy enabled stratification into low-, intermediate-, and high-risk groups, supporting a shift toward substrate-based risk assessment.

A different approach has been provided by radiomic texture analysis. In patients with DCM and severe systolic dysfunction, second-order features derived from grey-level co-occurrence matrices, including difference entropy, were associated with death or cardiac transplantation and remained predictive after adjustment for LGE extent and clinical variables [44]. Unlike first-order histogram entropy, these second-order measures incorporate relationships between neighbouring voxel intensities and therefore provide information about local textural organization. The two approaches should not be considered interchangeable.

Collectively, these studies suggest that LGE signal-intensity heterogeneity provides prognostic information in DCM beyond conventional measures of ventricular function and fibrosis burden. Both whole-LV and scar-specific first-order entropy have been associated with arrhythmic outcomes, while second-order texture measures have been linked to broader adverse outcomes.

### 7.2. Left Ventricular Non-Compaction

Left ventricular non-compaction (LVNC) is an uncommon myocardial phenotype characterized by a spongy-appearing myocardium, consisting of a thin or normal compacted epicardial layer overlying a prominent non-compacted endocardial layer with excessive trabeculations and deep intertrabecular recesses that communicate directly with the left ventricular cavity. The increasing use of CMR has markedly improved the detection of LVNC, but its clinical significance remains controversial, with ongoing debate over whether and in which patients it may represent a distinct cardiomyopathy, a morphological phenotype shared across a spectrum of myocardial diseases, or simply a normal variant [45]. LVNC has been associated with an increased risk of heart failure, thromboembolism and ventricular arrhythmias. However, the prognosis of LVNC remains remarkably heterogeneous and the risk stratification particularly challenging [46]. LGE presence has been associated with worse prognosis, but LGE relies on a subjective visual evaluation and might be erroneous when the degree of myocardial fibrosis is modest or when the myocardium is diffusely fibrotic [47]. This has prompted investigation of quantitative measures derived from the entire myocardial signal-intensity distribution (Table S4).

In a study by Ma et al. [48], left ventricular (LV) LGE entropy was significantly higher in LVNC patients who experienced MACEs than in those who remained event-free, but the composite was clinically broad, incorporating heart-failure events, ventricular and atrial arrhythmias, systemic embolic events, myocardial infarction, and cardiac death. The risk of MACE increased progressively with higher entropy values. Among the 30 observed events, heart failure was the most frequent component, while only nine were classified as arrhythmic events and one as cardiac death. Thus, the study demonstrates broader prognostic association rather than specific prediction of severe ventricular arrhythmia. Both LV entropy and left ventricular ejection fraction (LVEF) demonstrated good predictive performance, while a combined model incorporating both parameters achieved superior prognostic accuracy compared with either measure alone. These findings suggest that whole-LV entropy may provide information complementary to ventricular systolic function. However, the study was conducted at a single centre, included a relatively small number of events, and lacked comparison with native T1 mapping or extracellular volume; the authors therefore regarded the multivariable analysis as exploratory.

In a larger study by Gao et al., CMR-LGE-derived whole-LV entropy remained independently associated with a narrower but still mixed composite of cardiac death, ventricular arrhythmia requiring therapy, and heart-failure hospitalization after accounting for established imaging markers, including LVEF and LGE extent [49]. Notably, whole-LV entropy remained independently associated with MACEs in both LGE-positive and LGE-negative patients, suggesting potential prognostic value even in the absence of visually detectable LGE. Although this larger cohort and longer follow-up period strengthen the evidence, this was also a retrospective single-centre investigation without external validation or direct comparison with native T1 or extracellular volume.

Taken together, the LVNC studies support whole-LV entropy as a marker of overall cardiovascular risk, rather than specifically of hard arrhythmic risk. The two cohorts used composite endpoints that combined arrhythmic, heart-failure, mortality, and, in the Ma et al. [48] study, thromboembolic outcomes. This distinction is important because an association with MACEs does not establish an equivalent association with SCD, sustained VT/VF, or device-treated ventricular arrhythmia individually. Future LVNC studies should therefore examine these outcome domains separately to determine whether entropy predominantly reflects electrical instability, progressive ventricular dysfunction, or a broader adverse myocardial phenotype.

### 7.3. Autoimmune and Inflammatory Disease-Related Cardiomyopathy

Inflammatory disorders frequently involve the myocardium through a combination of inflammation, microvascular dysfunction, edema, and progressive fibrosis. However, myocardial involvement is often patchy or diffuse and may remain undetected by conventional CMR techniques until advanced stages of disease. Radiomic texture analysis has emerged as a promising approach to overcome these limitations by quantifying spatial heterogeneity within the myocardium (Table S5).

In patients with cardiac sarcoidosis, CMR-derived radiomic analysis demonstrated potential for distinguishing active from inactive disease. Although no individual radiomic feature achieved statistical significance, second-order texture features reflecting signal heterogeneity, particularly GLCM joint entropy, showed the best discriminatory performance. Machine-learning models incorporating radiomic signatures modestly improved classification accuracy. These findings suggest that myocardial tissue heterogeneity quantified from LGE-CMR may complement conventional imaging for assessing disease activity, although larger validation studies are needed before clinical implementation [50].

A different application has been investigated in systemic sclerosis, in which diffuse interstitial fibrosis may be difficult to characterize using conventional LGE alone. Native T1 mapping and extracellular volume fraction can identify interstitial expansion, whereas Yamamoto et al. evaluated the prognostic value of LGE-derived LV entropy together with these established tissue-characterization measures [51]. Higher LGE entropy was associated with adverse cardiovascular outcomes, including all-cause death, heart-failure hospitalization, and ventricular arrhythmia, and the combination of entropy and extracellular volume improved risk stratification. Entropy also provided further stratification among patients with relatively low extracellular volume, suggesting that the myocardial signal-intensity distribution may contain prognostic information complementary to measures of diffuse interstitial expansion. Importantly, however, this entropy measure should be interpreted as an assessment of signal-intensity heterogeneity rather than direct spatial organization of myocardial fibrosis. The small cohort and limited number of clinical events also restrict the strength and generalizability of these findings.

Taken together, inflammatory and autoimmune cardiomyopathies may represent a particularly distinctive application of entropy-based CMR analysis. Here, the value of entropy extends beyond conventional risk prediction toward the detection and phenotyping of subtle myocardial involvement that may be incompletely captured by standard imaging markers. Although the current evidence is still exploratory, it raises the possibility that entropy-based analysis could become most useful in these disorders not as a single universal biomarker but as a disease-specific tool tailored to the biological question—whether detecting occult involvement, assessing inflammatory activity, or refining prognosis.

## 8. Discussion

This review demonstrates that LGE entropy has emerged as a promising quantitative imaging biomarker capable of capturing myocardial tissue heterogeneity across a broad spectrum of cardiovascular diseases. Despite the heterogeneity of the diseases reviewed, including ischemic heart disease, hypertrophic cardiomyopathy, dilated cardiomyopathy, left ventricular non-compaction, and autoimmune and inflammatory cardiomyopathies, a remarkably consistent observation emerges: higher entropy is associated with worse clinical outcomes. The potential clinical value of entropy should be interpreted relative to established CMR markers. Across the available studies, entropy has shown associations that are partly independent of LGE extent, scar transmurality, grey-zone burden, and LVEF, while selected studies have demonstrated improved model discrimination or reclassification.

Across the reviewed studies, higher entropy has been associated with several distinct clinical outcome domains, including ventricular arrhythmias, device-treated ventricular arrhythmias, heart-failure-related events, mortality, and composite cardiovascular endpoints. These outcomes are not clinically interchangeable. Sustained VT/VF and SCD represent arrhythmic outcomes, appropriate ICD therapy is partly device- and programming-dependent, heart-failure hospitalization and cardiac transplantation reflect progressive pump failure, and all-cause mortality is etiologically nonspecific. Furthermore, the composition of MACEs varied substantially among studies. Despite this heterogeneity in outcome definitions, an emerging theme is the association of higher entropy with adverse cardiovascular phenotypes across multiple clinical domains. This consistency suggests that entropy may represent a disease-independent marker of adverse myocardial remodelling rather than a disease-specific imaging feature, although its prognostic significance is likely to vary according to the underlying disease substrate, region of interest, analytical method, and clinical endpoint.

An important distinction is that most evidence supporting the prognostic value of LGE entropy is based on first-order histogram entropy. Although second-order texture features derived from GLCM analysis have shown promising results, particularly in radiomics and machine-learning studies, their incremental clinical value beyond simpler first-order entropy remains uncertain and requires further investigation. Another important observation is that entropy may provide information complementary to conventional tissue-characterization techniques. While native T1 mapping, extracellular volume fraction, and conventional LGE quantify different aspects of myocardial pathology, first-order entropy characterizes the distribution and variability of signal-intensities within a predefined myocardial region without encoding their spatial arrangement. This may partly explain why entropy has remained independently associated with adverse outcomes after adjustment for established CMR parameters and, in some studies, has provided prognostic information even in patients with limited or absent visually detectable LGE. These findings suggest that signal-intensity heterogeneity may reflect aspects of the myocardial substrate not captured by fibrosis burden alone, although its biological correlates remain incompletely defined.

A central limitation of LGE entropy is that it is an image-derived measure of heterogeneity and not a direct measurement of myocardial tissue heterogeneity. First-order entropy is calculated from the distribution of signal-intensities within the segmented myocardium; therefore, increased entropy may reflect fibrosis heterogeneity, grey-zone scar, diffuse interstitial disease, or adverse remodelling, but may also reflect non-biological heterogeneity in the LGE image. This is particularly relevant because LGE-CMR analysis is affected by image noise, motion artefacts, and partial-volume effects, all of which can alter the apparent myocardial signal distribution and thereby increase measured entropy values [52]. Incomplete myocardial nulling, low signal-to-noise ratio, coil-related signal variation, blood-pool contamination, and imperfect segmentation may have similar effects.

Current studies have only partially addressed this issue. Antiochos et al. standardized LV entropy by magnetic field strength, acknowledging that field strength can affect signal-intensity distributions and measured entropy values, and excluded patients with uninterpretable LGE images [20]. However, most studies have not systematically reported image quality, signal-to-noise ratio, motion burden, denoising sensitivity, segmentation reproducibility, phantom calibration, or test–retest repeatability. Thus, elevated LGE entropy should currently be interpreted as a quantitative imaging biomarker that may reflect myocardial substrate complexity, rather than as a purely biological measurement of fibrosis heterogeneity.

Additional limitations remain. Most available studies are small, retrospective, and observational, with limited prospective multicentre validation. Differences in device programming and detection criteria further limit direct comparison of studies using ICD therapy as an endpoint. The histopathological correlates of LGE entropy remain incompletely defined, and first-order histogram entropy does not fully capture the three-dimensional spatial organization of scar. Validated clinical thresholds for entropy have not been established. The prognostic significance of serial changes in entropy during adverse remodelling, reverse remodelling, or response to medical therapy also remains unknown.

Future studies should standardize acquisition and post-processing methods, assess reproducibility across scanners and vendors, and determine whether entropy remains prognostically useful after accounting for image quality and acquisition-related signal variation. Importantly, entropy as a first-order measure quantifies signal-intensity distributions and does not directly encode the spatial arrangement. Its associations with arrhythmic outcomes should therefore be interpreted as evidence that signal-intensity heterogeneity may indirectly reflect an adverse myocardial substrate rather than as direct measurement of spatial scar complexity.

Recent developments in radiomics and artificial intelligence further enhance the potential clinical utility of entropy. Future studies should determine whether first-order histogram entropy is sufficient for clinical implementation or whether more computationally demanding second-order texture features provide meaningful incremental prognostic value. Rather than serving as an isolated biomarker, entropy may become an integral component of multiparametric imaging models combining clinical characteristics, ventricular function, parametric mapping, scar quantification, and additional radiomic features. Machine-learning algorithms are particularly well suited to exploit these multidimensional datasets and may enable more individualized prediction of arrhythmic risk, heart-failure progression, and therapeutic response. Future work should therefore focus on integrating entropy into comprehensive imaging-based risk prediction models rather than evaluating it in isolation.

## 9. Conclusions

LGE entropy is a promising imaging biomarker of myocardial tissue heterogeneity that provides independent—and possibly incremental—prognostic value beyond conventional CMR and LGE metrics and may play a central role in personalized risk stratification. Future studies are needed to validate its clinical utility, determine optimal analytical methods, and clarify how entropy metrics might be integrated into existing risk stratification algorithms.

## Data Availability

No new data were created or analyzed in this study. Data sharing is not applicable to this article.

## Supplementary Materials

The following supporting information can be downloaded at: https://www.mdpi.com/article/10.3390/diagnostics16172735/s1, Table S1. Characteristics and methodological approaches of LGE entropy studies in ischemic heart disease and myocardial infarction [28,29,30,31,32,33,34], Table S2. Characteristics and methodological approaches of LGE entropy studies in hypertrophic cardiomyopathy [9,25,26,27], Table S3. Characteristics of studies evaluating LGE-CMR-derived entropy and texture features in non-ischemic dilated cardiomyopathy [42,43,44], Table S4. Characteristics of studies evaluating LGE-CMR-derived entropy in left ventricular non-compaction [48,49], Table S5. Characteristics of CMR entropy and radiomic studies in autoimmune and inflammatory myocardial disease [50,51].

## References

[1] Materka A. (2004). Texture analysis methodologies for magnetic resonance imaging. Dialogues Clin. Neurosci..

[2] Castellano G., Bonilha L., Li L.M., Cendes F. (2004). Texture analysis of medical images. Clin. Radiol..

[3] Baessler B., Mannil M., Oebel S., Maintz D., Alkadhi H., Manka R. (2018). Subacute and chronic left ventricular myocardial scar: Accuracy of texture analysis on nonenhanced cine MR images. Radiology.

[4] Waugh S.A., Purdie C.A., Jordan L.B., Vinnicombe S., Lerski R.A., Martin P., Thompson A.M. (2016). Magnetic resonance imaging texture analysis classification of primary breast cancer. Eur. Radiol..

[5] Yokoo T., Wolfson T., Iwaisako K., Peterson M.R., Mani H., Goodman Z., Changchien C., Middleton M.S., Gamst A.C., Mazhar S.M. (2015). Evaluation of Liver Fibrosis Using Texture Analysis on Combined-Contrast-Enhanced Magnetic Resonance Images at 3.0T. Biomed. Res. Int..

[6] Gräni C., Eichhorn C., Bière L., Kaneko K., Murthy V.L., Agarwal V., Aghayev A., Steigner M., Blankstein R., Jerosch-Herold M. (2019). Comparison of myocardial fibrosis quantification methods by cardiovascular magnetic resonance imaging for risk stratification of patients with suspected myocarditis. J. Cardiovasc. Magn. Reson..

[7] Scott P.A., Rosengarten J.A., Murday D.C., Peebles C.R., Harden S.P., Curzen N.P., Morgan J.M. (2013). Left ventricular scar burden specifies the potential for ventricular arrhythmogenesis: An LGE-CMR study. J. Cardiovasc. Electrophysiol..

[8] McAlindon E., Pufulete M., Lawton C., Angelini G.D., Bucciarelli-Ducci C. (2015). Quantification of infarct size and myocardium at risk: Evaluation of different techniques and its implications. Eur. Heart J. Cardiovasc. Imaging.

[9] Ye Y., Ji Z., Zhou W., Pu C., Li Y., Zhou C., Hu X., Chen C., Sun Y., Huang Q. (2021). Mean scar entropy by late gadolinium enhancement cardiac magnetic resonance is associated with ventricular arrhythmias events in hypertrophic cardiomyopathy. Front. Cardiovasc. Med..

[10] Balaban G., Halliday B.P., Bai W., Porter B., Malvuccio C., Lamata P., Rinaldi C.A., Plank G., Rueckert D., Prasad S.K. (2019). Scar shape analysis and simulated electrical instabilities in a non-ischemic dilated cardiomyopathy patient cohort. PLoS Comput. Biol..

[11] Heidary S., Patel H., Chung J., Yokota H., Gupta S.N., Bennett M.V., Katikireddy C., Nguyen P., Pauly J.M., Terashima M. (2010). Quantitative tissue characterization of infarct core and border zone in patients with ischemic cardiomyopathy by magnetic resonance is associated with future cardiovascular events. J. Am. Coll. Cardiol..

[12] Zeidan-Shwiri T., Yang Y., Lashevsky I., Kadmon E., Kagal D., Dick A., Farkash A.L., Paul G., Gao D., Shurrab M. (2015). Magnetic resonance estimates of the extent and heterogeneity of scar tissue in ICD patients with ischemic cardiomyopathy predict ventricular arrhythmia. Heart Rhythm..

[13] Gao P., Yee R., Gula L., Krahn A.D., Skanes A., Leong-Sit P., Klein G.J., Stirrat J., Fine N., Pallaveshi L. (2012). Prediction of arrhythmic events in ischemic and dilated cardiomyopathy patients referred for implantable cardiac defibrillator: Evaluation of multiple scar quantification measures for late gadolinium enhancement magnetic resonance imaging. Circ. Cardiovasc. Imaging.

[14] Roes S.D., Borleffs C.J.W., van der Geest R.J., Westenberg J.J., Marsan N.A., Kaandorp T.A., Reiber J.H., Zeppenfeld K., Lamb H.J., de Roos A. (2009). Infarct tissue heterogeneity assessed with contrast-enhanced MRI predicts spontaneous ventricular arrhythmia in patients with ischemic cardiomyopathy and implantable cardioverter-defibrillator. Circ. Cardiovasc. Imaging.

[15] Schmidt A., Azevedo C.F., Cheng A., Gupta S.N., Bluemke D.A., Foo T.K., Gerstenblith G., Weiss R.G., Marba E., Tomaselli G.F. (2007). Infarct tissue heterogeneity by magnetic resonance imaging identifies enhanced cardiac arrhythmia susceptibility in patients with left ventricular dysfunction. Circulation.

[16] Zahid S., Cochet H., Boyle P.M., Schwarz E.L., Whyte K.N., Vigmond E.J., Dubois R., Hocini M., Haïssaguerre M., Jaïs P. (2016). Patient-derived models link re-entrant driver localization in atrial fibrillation to fibrosis spatial pattern. Cardiovasc. Res..

[17] Ali R.L., Hakim J.B., Boyle P.M., Zahid S., Sivasambu B., Marine J.E., Calkins H., Trayanova N.A., Spragg D.D. (2019). Arrhythmogenic propensity of the fibrotic substrate after atrial fibrillation ablation: A longitudinal study using magnetic resonance imaging-based atrial models. Cardiovasc. Res..

[18] Suzuki Y., Kiuchi K., Takami M., Imamura K., Sakai J., Nakamura T., Yatomi A., Sonoda Y., Takahara H., Nakasone K. (2025). Late gadolinium enhancement in areas with electrically fractionated potentials during sinus rhythm in patients with atrial fibrillation. Heart Vessels.

[19] Pham V.-T., Lin C., Tran T.-T., Su M.-Y.M., Lin Y.-K., Nien C.-T., Tseng W.-Y.I., Lin J.-L., Lo M.-T., Lin L.-Y. (2020). Predicting ventricular tachyarrhythmia in patients with systolic heart failure based on texture features of the gray zone from contrast-enhanced magnetic resonance imaging. J. Cardiol..

[20] Antiochos P., Ge Y., van der Geest R.J., Madamanchi C., Qamar I., Seno A., Jerosch-Herold M., Tedrow U.B., Stevenson W.G., Kwong R.Y. (2022). Entropy as a measure of myocardial tissue heterogeneity in patients with ventricular arrhythmias. JACC Cardiovasc. Imaging.

[21] Zhao X., Song Y., Zhang L., Wang L., Zhao X., Wang J. (2025). Cardiac Magnetic Resonance-Derived Left Ventricular Mechanical Dispersion Improves Risk Stratification of Hypertrophic Cardiomyopathy and its Correlations with Scar Heterogeneity. J. Cardiovasc. Transl. Res..

[22] Gould J., Porter B., Claridge S., Chen Z., Sieniewicz B.J., Sidhu B.S., Niederer S., Bishop M.J., Murgatroyd F., Ganeshan B. (2019). Mean entropy predicts implantable cardioverter-defibrillator therapy using cardiac magnetic resonance texture analysis of scar heterogeneity. Heart Rhythm..

[23] Gould J., Porter B., Sidhu B.S., Claridge S., Chen Z., Sieniewicz B.J., Elliott M., Mehta V., Campos F.O., Bishop M.J. (2020). High mean entropy calculated from cardiac MRI texture analysis is associated with antitachycardia pacing failure. Pacing Clin. Electrophysiol..

[24] Zhang Y., Wang L., Wang J., Zhao X. (2025). Increased Entropy Predicts Adverse Cardiac Events in Patients with High Cardiovascular Risk and Hypertension: A Novel Imaging Parameter Derived from Late Gadolinium Enhancement. Rev. Cardiovasc. Med..

[25] Antiochos P., Ge Y., Jerosch-Herold M., David L.-P., Heydari B., Kolm P., Kim D.-Y., van der Geest R.J., Watkins H., Desai M.Y. (2025). Myocardial entropy and risk predictors in hypertrophic cardiomyopathy: An analysis from the NHLBI HCM registry. Circ. Cardiovasc. Imaging.

[26] Zhao X., Jin F., Wang J., Zhao X., Wang L., Wei H. (2023). Entropy of left ventricular late gadolinium enhancement and its prognostic value in hypertrophic cardiomyopathy a new CMR assessment method. Int. J. Cardiol..

[27] Gu Z.-Y., Qian Y.-F., Chen B.-H., Wu C.-W., Zhao L., Xue S., Wu L.-M., Wang Y.-Y. (2023). Late gadolinium enhancement entropy as a new measure of myocardial tissue heterogeneity for prediction of adverse cardiac events in patients with hypertrophic cardiomyopathy. Insights Imaging.

[28] Androulakis A.F., Zeppenfeld K., Paiman E.H., Piers S.R., Wijnmaalen A.P., Siebelink H.-M.J., Sramko M., Lamb H.J., van der Geest R.J., de Riva M. (2019). Entropy as a Novel Measure of Myocardial Tissue Heterogeneity for Prediction of Ventricular Arrhythmias and Mortality in Post-Infarct Patients. JACC Clin. Electrophysiol..

[29] Wang L., Peng L., Zhao X., Ma Y., Jin F., Zhao X. (2023). Prognostic Value of Entropy Derived from Late Gadolinium Enhancement Images to Adverse Cardiac Events in Post-Myocardial Infarction Patients. Acad. Radiol..

[30] Zhao X., Zhao X., Jin F., Wang L., Zhang L. (2023). Prognostic Value of Cardiac-MRI Scar Heterogeneity Combined with Left Ventricular Strain in Patients with Myocardial Infarction. J. Magn. Reson. Imaging.

[31] Zhao X., Zhang L., Wang L., Zhang W., Song Y., Zhao X., Li Y. (2025). Magnetic resonance imaging quantification of left ventricular mechanical dispersion and scar heterogeneity optimize risk stratification after myocardial infarction. BMC Cardiovasc. Disord..

[32] Wang W.-X., Gao Y., Wang J., Liu M.-X., Gu H., Yuan X.-S., Wang X.-M. (2024). Left ventricular entropy is a novel predictor of major adverse cardiac events (MACE) in patients with coronary atherosclerosis: A multi-center study. Eur. Radiol..

[33] Li B., Wang W., Gao Y., Wang J., Zhu R., Yuan X., Zhang S., Wang X., Gu H. (2025). Prognostic Value of Late Gadolinium Enhancement Cardiac MRI-derived Entropy in Patients after Myocardial Infarction. Radiol. Cardiothorac. Imaging.

[34] Zaidi H.A., Jones R.E., Hammersley D.J., Hatipoglu S., Balaban G., Mach L., Halliday B.P., Lamata P., Prasad S.K., Bishop M.J. (2023). Machine learning analysis of complex late gadolinium enhancement patterns to improve risk prediction of major arrhythmic events. Front. Cardiovasc. Med..

[35] Cui H., Schaff H.V., Carvalho J.L., Nishimura R.A., Geske J.B., Dearani J.A., Lahr B.D., Lee A.T., Bos J.M., Ackerman M.J. (2021). Myocardial histopathology in patients withobstructive hypertrophic cardiomyopathy. J. Am. Coll. Cardiol..

[36] Ariga R., Tunnicliffe E.M., Manohar S.G., Mahmod M., Raman B., Piechnik S.K., Francis J.M., Robson M.D., Neubauer S., Watkins H. (2019). Identification of Myocardial Disarray in Patients with Hypertrophic Cardiomyopathy and Ventricular Arrhythmias. J. Am. Coll. Cardiol..

[37] Díez J., González A., Kovacic J.C. (2020). Myocardial interstitial fibrosis in nonischemic heart disease, part 3/4: JACC focus seminar. J. Am. Coll. Cardiol..

[38] Galati G., Leone O., Pasquale F., Olivotto I., Biagini E., Grigioni F., Pilato E., Lorenzini M., Corti B., Foà A. (2016). Histological and Histometric Characterization of Myocardial Fibrosis in End-Stage Hypertrophic Cardiomyopathy: A Clinical-Pathological Study of 30 Explanted Hearts. Circ. Heart Fail..

[39] Wu K.C. (2017). Sudden cardiac death substrate imaged by magnetic resonance imaging: From investigational tool to clinical applications. Circ. Cardiovasc. Imaging.

[40] Halliday B.P., Cleland J.G.F., Goldberger J.J., Prasad S.K. (2017). Personalizing risk stratification for sudden death in dilated cardiomyopathy: The past, present, and future. Circulation.

[41] Køber L., Thune J.J., Nielsen J.C., Haarbo J., Videbæk L., Korup E., Jensen G., Hildebrandt P., Steffensen F.H., Bruun N.E. (2016). Defibrillator Implantation in Patients with Nonischemic Systolic Heart Failure. N. Engl. J. Med..

[42] Muthalaly R.G., Kwong R.Y., John R.M., van der Geest R.J., Tao Q., Schaeffer B., Tanigawa S., Nakamura T., Kaneko K., Tedrow U.B. (2019). Left ventricular entropy is a novel predictor of arrhythmic events in patients with dilated cardiomyopathy receiving defibrillators for primary prevention. JACC Cardiovasc. Imaging.

[43] Hammersley D.J., Zaidi H.A., Jones R.E., Hatipoglu S., Androulakis E., Balaban G., Mach L., Lota A.S., Khalique Z., De Marvao A. (2025). Fibrosis Entropy Is Associated With Life-Threatening Arrhythmia in Nonischemic Cardiomyopathy. J. Am. Heart Assoc..

[44] Shu S., Wang C., Hong Z., Zhou X., Zhang T., Peng Q., Wang J., Zheng C. (2021). Prognostic value of late enhanced cardiac magnetic resonance imaging derived texture features in dilated cardiomyopathy patients with severely reduced ejection fractions. Front. Cardiovasc. Med..

[45] Martínez-Tittonel L.E., Ciorba F.R., Bayona-Huguet X., Kaplinsky E. (2025). Left Ventricular Non-Compaction Cardiomyopathy: A Review of the Pathophysiology, Epidemiology, Diagnosis, Genetics, and Clinical Management. J. Pers. Med..

[46] Casas G., Limeres J., Oristrell G., Gutierrez-Garcia L., Andreini D., Borregan M., Larrañaga-Moreira J.M., Lopez-Sainz A., Codina-Solà M., Teixido-Tura G. (2021). Clinical risk prediction in patients with left ventricular myocardial noncompaction. J. Am. Coll. Cardiol..

[47] Iles L.M., Ellims A.H., Llewellyn H., Hare J.L., Kaye D.M., McLean C.A., Taylor A.J. (2015). Histological validation of cardiac magnetic resonance analysis of regional and diffuse interstitial myocardial fibrosis. Eur. Heart J. Cardiovasc. Imaging.

[48] Ma Y.-T., Wang L.-J., Zhao X.-Y., Zheng Y., Sha L.-H., Zhao X.-X. (2023). Can left ventricular entropy by cardiac magnetic resonance late gadolinium enhancement be a prognostic predictor in patients with left ventricular non-compaction?. Diagn. Interv. Radiol..

[49] Gao Y., Liu M., Ju Z., Wang H., Gu H., Wang X. (2023). Entropy as a novel predictor of cardiovascular events in patients with left ventricular noncompaction. Int. J. Cardiol..

[50] Mushari N.A., Soultanidis G., Duff L., Trivieri M.G., Fayad Z.A., Robson P.M., Tsoumpas C. (2023). Exploring the utility of cardiovascular magnetic resonance radiomic feature extraction for evaluation of cardiac sarcoidosis. Diagnostics.

[51] Yamamoto A., Nagao M., Shirai Y., Nakao R., Sakai A., Kaneko K., Arashi H., Minami Y., Sakai S., Yamaguchi J. (2023). Cardiac magnetic resonance imaging T1 mapping and late gadolinium enhancement entropy: Prognostic value in patients with systemic sclerosis. J. Cardiol..

[52] Xing J., Wang S., Bilchick K.C., Patel A.R., Zhang M. Joint deep learning for improved myocardial scar detection from cardiac MRI. Proceedings of the 2023 IEEE 20th International Symposium on Biomedical Imaging (ISBI).

