# Supplementary material for: Late Gadolinium Enhancement Entropy as a Novel Imaging Biomarker of Myocardial Tissue Heterogeneity—A Comprehensive Review
Source: Diagnostics (Basel). 2026 Aug 26;16(17):2735. doi: 10.3390/diagnostics16172735 (PMC13565341; doi:10.3390/diagnostics16172735)
Supplement: Supplementary file 1 [file diagnostics-16-02735-s001.zip › Table S1 Ischemic LGE studies_.pdf]

Table S1. Characteristics and methodological approaches of LGE entropy studies in ischaemic heart disease and myocardial infarction

First author, year	Study design	Sample size; events	Underlying cardiac disease	Field strength	Entropy / texture metric	Region of interest	Segmentation method	Primary endpoint	Follow-up
Androulakis, 2019 [28]	Retrospective, single-center cohort; consecutive post-MI patients undergoing LGE-CMR before ICD implantation	154; 46 appropriate ICD therapies; 41 deaths	Prior MI / ischemic cardiomyopathy; primary- or secondary-prevention ICD cohort	1.5 T	First-order Shannon histogram entropy	Total infarct scar and whole-LV myocardium; core and grey zone quantified as conventional comparators	Manual endocardial/epicardial contours; semi-automated SI thresholds: total scar >35% maximum SI, grey zone 35–<50%, core ≥50%; entropy calculated automatically in MATLAB	Appropriate ICD therapy for monomorphic VT or VF; all-cause mortality	Median 56 months (IQR 40–73)
Wang, 2023 [29]	Retrospective, single-center observational cohort	84; 33 MACE (6 cardiac deaths, 10 HF, 5 recurrent MI, 4 strokes, 2 VA, 6 severe-angina rehospitalizations)	Previous MI with positive ischemic-pattern LGE	3.0 T	First-order Shannon histogram entropy	Infarct core (IC), border zone (BZ), combined infarct and border zone (IBZ), and whole LV	Manual LV contours; semi-automated n-SD segmentation in CVI42: IC >3 SD, BZ 2–3 SD, IBZ ≥2 SD above remote myocardium	Composite MACE: severe-angina rehospitalization, HF, stroke, recurrent nonfatal MI, VA, or cardiac death	Median 19 months
Zhao, 2023 [30]	Prospective, single-center observational cohort	157; 26 primary-endpoint events, 30 secondary-endpoint events, and 43 patients with either or both endpoints	Previous MI (>4 months) with ischemic-pattern LGE; no prior sustained VA	3.0 T	First-order Shannon histogram entropy derived from voxel SI probability distributions	Border zone (BZ), infarct core (IC), and combined infarct core plus border zone (IBZ)	Manual endocardial/epicardial contours on infarcted short-axis LGE slices in CVI42; remote myocardium selected manually; IC ≥3 SD above remote myocardium, BZ 2–<3 SD, and IBZ = BZ + IC; Python-based entropy calculation	Primary: SCD, sustained VA, or ICD therapy for VA. Secondary: all-cause mortality, recurrent MI, or new HF	Median 2.8 years (range 17–52 months)
Zhao, 2025 [31]	Retrospective, single-center cohort of consecutive post-MI patients	181; 42 composite events	Previous MI (>3 months)	3.0 T	First-order histogram entropy calculated from voxel SI probability distributions	BZ, IC, IBZ, and whole LV	Manual LV and remote-myocardium contours; SD thresholds (IC >3 SD, IBZ >2 SD, BZ 2–3 SD); semi-automated infarct detection with manual correction; Python-based entropy calculation	Composite of SCD, sustained VA and/or ICD therapy, and new HF requiring readmission	31 months (reported as median in the Abstract and mean in the Results)
Wang, 2024 [32]	Retrospective, two-center observational cohort	314; 110 MACE	Coronary artery disease; 229 (72.9%) had prior MI	1.5 T and 3.0 T	First-order Shannon histogram entropy; values standardized by field strength	Whole-LV myocardium	Semi-automated endocardial/epicardial contours on short-axis LGE in ResearchMass with blood-pool exclusion; LGE burden quantified separately by FWHM	Composite MACE: SCD or rehospitalization for severe angina, HF, recurrent nonfatal MI, or VA	Median 13 months
Li, 2025 [33]	Retrospective, two-center observational cohort with an independent second-center validation set	226 derivation cohort, 69 validation cohort; derivation cohort: 72 MACE including 19 VA	Previous MI with positive LGE	3.0 T at both centers	First-order Shannon histogram entropy derived from pixel SI probability distributions	Whole-LV myocardium and total scar tissue	Semi-automated endocardial/epicardial contours on short-axis LGE in ResearchMass with blood-pool exclusion; scar defined semi-automatically as SI >35% of maximum SI; LGE extent quantified separately by FWHM; manual correction by experienced CMR readers	Primary: MACE (SCD, VA requiring therapy, HF rehospitalization, recurrent nonfatal MI, or severe-angina rehospitalization). Secondary: VA	Median 21 months (IQR 15–31)
Zaidi, 2023 [34]	Prospective, single-center CMR registry analysis with machine-learning modelling	397; 55 major arrhythmic events	Stable coronary artery disease with LGE; 302 (76.1%) had prior MI	1.5 T	Standard Shannon entropy plus scar morphology/texture metrics (e.g., gradient, local binary patterns, interface area, components)	Core infarct, peri-infarct zone (PIZ), and combined core + PIZ; slice features aggregated across the LV	Manual LV contours and expert-defined core/PIZ/remote regions; FWHM and 2–3 SD / 2–5 SD methods; 10% endocardial and epicardial erosion	Major arrhythmic event: SCD, aborted SCD, or haemodynamically unstable VT	Median 6 years

Abbreviations: BZ, border zone; CAD, coronary artery disease; CMR, cardiac magnetic resonance; FWHM, full width at half maximum; HF, heart failure; IBZ, infarct core plus border zone; IC, infarct core; ICD, implantable cardioverter-defibrillator; IQR, interquartile range; LGE, late gadolinium enhancement; LV, left ventricle/left ventricular; MACE, major adverse cardiovascular event(s); MI, myocardial infarction; PIZ, peri-infarct zone; PSIR, phase-sensitive inversion recovery; SD, standard deviation; SCD, sudden cardiac death; SI, signal intensity; VA, ventricular arrhythmia; VF, ventricular fibrillation; VT, ventricular tachycardia.
