# Supplementary material for: Late Gadolinium Enhancement Entropy as a Novel Imaging Biomarker of Myocardial Tissue Heterogeneity—A Comprehensive Review
Source: Diagnostics (Basel). 2026 Aug 26;16(17):2735. doi: 10.3390/diagnostics16172735 (PMC13565341; doi:10.3390/diagnostics16172735)
Supplement: Supplementary file 1 [file diagnostics-16-02735-s001.zip › Table S2 HCM LGE Studies_.pdf]

Table S2. Characteristics and methodological approaches of LGE entropy studies in hypertrophic cardiomyopathy

First author, year	Study design	Sample size; events	Underlying cardiac disease	Field strength	Entropy / texture metric	Region of interest	Segmentation method	Primary endpoint	Follow-up
Ye, 2021 [9]	Retrospective, single-center observational cohort; HCM patients with visible LGE and available 24-h ambulatory ECG	68; 31 with VA (4 aborted cardiac arrest, 7 sustained VT, 20 NSVT)	HCM with visible LGE	1.5 T (Siemens and GE)	First-order Shannon histogram entropy (log2); SI normalized to 0-1024 and graded from 0 to 10	LGE-defined scar and whole-LV myocardium	Manual endocardial, epicardial, and scar contours; LGE defined as >=5 SD above remote myocardium; PyRadiomics/Python entropy; second-reader reproducibility	Presence of VA: aborted cardiac arrest, sustained VT, or NSVT on 24-h ambulatory ECG and clinical records	Median 25 months (IQR reported as 13-13)
Antiochos, 2025 [25]	Cross-sectional analysis within the prospective, international, multicenter NHLBI HCM Registry	1,736 entropy cohort; 1,630 with Holter; 233 with sustained or NSVT; 858 without-LGE subgroup	HCM across a broad risk spectrum	1.5 T and 3.0 T; Siemens, Philips, and GE	First-order Shannon histogram entropy (log2); values adjusted for site, field strength, and vendor	Whole-LV myocardium	Manual/verified endocardial and epicardial contours imported from registry LGE analysis; automated entropy calculation in Mass Research software	Cross-sectional presence of sustained or non-sustained VT on ambulatory Holter; associations with ESC SCD risk score and other biomarkers	Not applicable; prospective clinical-event follow-up was ongoing
Zhao, 2023 [26]	Prospective, single-center cohort of consecutive adults	109; 21 composite events (3 SCD, 9 ICD treatments for VA, 9 new HF)	HCM	3.0 T (Philips Achieva)	First-order Shannon histogram entropy; SI normalized to 0-1024 and graded from 0 to 10	Whole-LV myocardium	Manual endocardial/epicardial contours on all short-axis LGE images by two radiologists; automated voxel-SI extraction/Python entropy; LGE burden by 6-SD remote-myocardium method	Composite of SCD, new HF/hospitalization, or ICD treatment for VA	Mean 23 +/- 7 months
Gu, 2023 [27]	Retrospective observational cohort	337; 33 primary HF readmissions; 43 secondary events (33 HF, 8 cardiovascular deaths, 2 non-CV deaths)	HCM	3.0 T (Siemens and Philips)	First-order whole-LV histogram entropy calculated in MATLAB; equation as reported by the authors	Whole-LV myocardium	Manual endocardial/epicardial contours on LGE images with blood-pool exclusion; automated entropy calculation; LGE extent quantified separately by FWHM in CVI42	Primary: HF readmission. Secondary: HF readmission plus cardiac/SCD and non-CV death, as reported	Median 24 months (reported as 24 +/- 13 months)

Abbreviations: AHA/ACC, American Heart Association/American College of Cardiology; CMR, cardiac magnetic resonance; ECG, electrocardiography; ESC, European Society of Cardiology; FWHM, full width at half maximum; HCM, hypertrophic cardiomyopathy; HF, heart failure; ICD, implantable cardioverter-defibrillator; IQR, interquartile range; LGE, late gadolinium enhancement; LV, left ventricle/left ventricular; NHLBI HCMR, National Heart, Lung, and Blood Institute Hypertrophic Cardiomyopathy Registry; NSVT, non-sustained ventricular tachycardia; PSIR, phase-sensitive inversion recovery; ROI, region of interest; SCD, sudden cardiac death; SD, standard deviation; SI, signal intensity; VA, ventricular arrhythmia; VT, ventricular tachycardia.
