# Supplementary material for: Late Gadolinium Enhancement Entropy as a Novel Imaging Biomarker of Myocardial Tissue Heterogeneity—A Comprehensive Review
Source: Diagnostics (Basel). 2026 Aug 26;16(17):2735. doi: 10.3390/diagnostics16172735 (PMC13565341; doi:10.3390/diagnostics16172735)
Supplement: Supplementary file 1 [file diagnostics-16-02735-s001.zip › Table S3 DCM LGE Entropy Studies_.pdf]

Table S3. Characteristics of studies evaluating LGE-CMR-derived entropy and texture features in non-ischemic dilated cardiomyopathy

First author, year	Study design	Sample size / events	Disease	Field strength	Entropy type	Region of interest	Segmentation / processing	Primary endpoint	Follow-up
Muthalaly et al., 2019 [42]	Registry-based observational cohort; consecutive DCM patients receiving primary-prevention ICDs	N=130; 18 arrhythmic events; 17 end-stage HF events; 7 deaths	DCM	3.0 T Siemens / 1.5 T GE (101 / 29 patients)	First-order Shannon histogram entropy	Whole LV myocardium across the LGE short-axis stack	LV contours traced on LGE short-axis images; entropy calculated semi-automatically from the aggregated myocardial SI histogram across all slices; FWHM used separately for dense-scar mass; field-strength correction applied	Arrhythmic events: appropriate ICD therapy, sustained ventricular arrhythmia, or SCD	Median 3.2 years
Hammersley et al., 2025 [43]	Prospective observational cohort; LGE-positive NICM with LGE-negative comparator*	291 LGE(+) NICM (250 DCM or NDLVC); 38 LTA; 574 LGE(−) NICM comparator	NICM, predominantly DCM (85.9%)*	1.5 T Siemens Sonata/Avanto	First-order Shannon entropy of fibrosis SI; per-slice values aggregated across the LV stack	LGE-defined core fibrosis, GZ fibrosis, and combined core+GZ fibrosis	Semi-automatic LV contours in CVI42; core fibrosis ≥50% of maximal SI (FWHM); GZ ≥35% to <50%; proprietary software used for fibrosis entropy calculation	LTA: SCD, aborted SCD, or sustained VT	Median 6.3 years (IQR 4.6–9.1); follow-up truncated at 10 years
Shu et al., 2021 [44]	Retrospective, single-center observational cohort; DCM with LVEF <35%	N=114; 29 endpoints (12 deaths, 17 cardiac transplantations)	DCM with severe systolic dysfunction	1.5 T or 3.0 T Siemens MAGNETOM Aera/Skyra	Second-order GLCM texture features, including GLCM difference entropy	Whole LV myocardium on late-enhanced continuous short-axis images	LV myocardial contours from conventional analysis; PyRadiomics; gray values normalized to 0–255 and discretized (bin width 5) into 52 gray levels; LGE extent defined separately at >6 SD above remote myocardium	Composite of all-cause death or cardiac transplantation	Median 504.5 days

*Hammersley et al. studied LGE-positive nonischemic cardiomyopathy: 250/291 patients (85.9%) had DCM and 41/291 (14.1%) had NDLVC; a separate LGE-negative NICM comparator cohort (n=574) was included in survival analyses.

Abbreviations: CMR, cardiac magnetic resonance; DCM, dilated cardiomyopathy; GLCM, gray-level co-occurrence matrix; GZ, gray zone; HF, heart failure; ICD, implantable cardioverter-defibrillator; LGE, late gadolinium enhancement; LTA, life-threatening arrhythmia; LV, left ventricle; LVEF, left ventricular ejection fraction; NDLVC, Non-dilated left ventricular cardiomyopathy; NICM, nonischemic cardiomyopathy; PSIR, phase-sensitive inversion recovery; SCD, sudden cardiac death; SI, signal intensity; VT, ventricular tachycardia.
