# Supplementary material for: Late Gadolinium Enhancement Entropy as a Novel Imaging Biomarker of Myocardial Tissue Heterogeneity—A Comprehensive Review
Source: Diagnostics (Basel). 2026 Aug 26;16(17):2735. doi: 10.3390/diagnostics16172735 (PMC13565341; doi:10.3390/diagnostics16172735)
Supplement: Supplementary file 1 [file diagnostics-16-02735-s001.zip › Table S4 LVNC LGE Studies_.pdf]

Table S4. Characteristics of studies evaluating LGE-CMR-derived entropy in left ventricular non-compaction

First author, year	Study design	Sample size / events	Disease	Field strength	Entropy type	Region of interest	Segmentation / processing	Primary endpoint	Follow-up
Ma et al., 2023 [48]	Single-center observational cohort; consecutive patients; study registered (CTR2200062045)	N=86; 30 MACE (16 HF, 9 arrhythmia, 2 stroke, 2 MI, 1 cardiac death)	LVNC	3.0 T Philips Achieva	First-order Shannon histogram entropy	Whole LV myocardium on short-axis LGE	LV endocardial/epicardial contours semi-automatically outlined in CVI42; LGE quantified at >6 SD above reference myocardium; images exported to Python; SI discretized 0–255 and entropy calculated from the SI probability distribution	MACE: HF hospitalization/CRT/heart transplantation; arrhythmia (VF, sustained or NSVT, ICD, AF); systemic embolism/stroke/MI/peripheral embolism; cardiac death	Median 18 months
Gao et al., 2023 [49]	Retrospective single-center cohort; consecutive patients	N=143; 42 MACE (25 HF hospitalizations, 12 ventricular arrhythmias, 5 cardiac deaths)	LVNC	3.0 T Siemens MAGNETOM Prisma	First-order Shannon histogram entropy	Whole LV myocardium on PSIR LGE short-axis stack	Manual epicardial/endocardial contours; blood-pool signal excluded; entropy automatically generated in ResearchMass from SI probability distribution; LGE extent quantified separately by FWHM	MACE: cardiac death, ventricular arrhythmia requiring therapy, or HF hospitalization/need for cardiac therapy	Median 38 months (IQR 18–58; ≈3.2 years)

Abbreviations: AF, atrial fibrillation; CMR, cardiac magnetic resonance; CRT, cardiac resynchronization therapy; FWHM, full width at half maximum; HF, heart failure; ICD, implantable cardioverter-defibrillator; LGE, late gadolinium enhancement; LV, left ventricle; LVNC, left ventricular non-compaction; MACE, major adverse cardiovascular event; MI, myocardial infarction; NSVT, non-sustained ventricular tachycardia; PSIR, phase-sensitive inversion recovery; SI, signal intensity; VF, ventricular fibrillation.
