# Supplementary material for: Late Gadolinium Enhancement Entropy as a Novel Imaging Biomarker of Myocardial Tissue Heterogeneity—A Comprehensive Review
Source: Diagnostics (Basel). 2026 Aug 26;16(17):2735. doi: 10.3390/diagnostics16172735 (PMC13565341; doi:10.3390/diagnostics16172735)
Supplement: Supplementary file 1 [file diagnostics-16-02735-s001.zip › Table S5 Autoimmune Inflammatory CMR Studies_.pdf]

Table S5. Characteristics of CMR entropy and radiomic studies in autoimmune and inflammatory myocardial disease

First author, year	Study design	Sample size / events	Disease	Field strength	Entropy / radiomic type	Region of interest	Segmentation / processing	Primary endpoint / application	Follow-up
Mushari et al., 2023 [50]	Single-center exploratory radiomics study; secondary analysis of PET-CMR data	N=61; 30 active CS and 31 inactive CS; no longitudinal events assessed	Cardiac sarcoidosis	Single Siemens Biograph mMR PET-CMR system; field strength not explicitly stated	Second-order GLCM joint entropy within a 94-feature radiomic panel; additional first- and higher-order features	Entire LV myocardium on LGE-CMR	Epicardial/endocardial boundaries drawn in 3D Slicer; PyRadiomics v3.0.1; 94 features; default parameters; no harmonization required because data came from one scanner	Discrimination of active vs inactive CS using PET-CMR classification (FDG uptake as activity reference); no prognostic endpoint	Not applicable (cross-sectional classification study)
Yamamoto et al., 2023 [51]	Retrospective, single-center prognostic cohort	N=24; 5 patients with MACE (1 death, 4 HF hospitalizations, 1 sustained VT; events may overlap)	Systemic sclerosis	3.0 T Philips Ingenia	First-order LGE histogram entropy derived from the myocardial pixel signal-intensity distribution	LV myocardium on a mid-LV short-axis LGE image	Manual epicardial/endocardial contours; SYNAPSE VINCENT used to calculate entropy from the LGE signal-intensity histogram; LGE presence assessed visually and extent by 17-segment model	MACE: all-cause death, HF hospitalization, sustained VT, or ventricular fibrillation	27 +/- 14 months

Mushari et al. used a single integrated Siemens Biograph mMR PET-CMR system; the MR field strength was not explicitly stated in the article.

Abbreviations: CMR, cardiac magnetic resonance; CS, cardiac sarcoidosis; ECV, extracellular volume; FDG, fluorodeoxyglucose; GLCM, gray-level co-occurrence matrix; HF, heart failure; LGE, late gadolinium enhancement; LV, left ventricle; MACE, major adverse cardiac event; PET, positron emission tomography; PSIR, phase-sensitive inversion recovery; ROI, region of interest; SSc, systemic sclerosis; VT, ventricular tachycardia.
